# Using mHealth to Support Postabortion Contraceptive Use: Results From a Feasibility Study in Urban Bangladesh

**DOI:** 10.2196/formative.5151

**Published:** 2017-10-27

**Authors:** Kamal Kanti Biswas, Altaf Hossain, Rezwana Chowdhury, Kathryn Andersen, Sharmin Sultana, S M Shahidullah, Erin Pearson

**Affiliations:** ^1^ Ipas Bangladesh Dhaka Bangladesh; ^2^ Bangladesh Association for Prevention of Septic Abortion Dhaka Bangladesh; ^3^ Ipas Chapel Hill, NC United States; ^4^ Department of Global Health and Population Harvard T.H. Chan School of Public Health Boston, MA United States

**Keywords:** mHealth, Bangladesh, contraceptive usage, postabortion contraception

## Abstract

**Background:**

As access to mobile technology improves in low- and middle-income countries, it becomes easier to provide information about sensitive issues, such as contraception and abortion. In Bangladesh, 97% of the population has access to a mobile signal, and the equity gap is closing in mobile phone ownership. Bangladesh has a high pregnancy termination rate and improving effective use of contraception after abortion is essential to reducing subsequent unwanted pregnancies.

**Objective:**

This study examines the feasibility and acceptability of implementing a short message service (SMS) text message-based mHealth intervention to support postabortion contraceptive use among abortion clients in Bangladesh, including women’s interest in the intervention, intervention preferences, and privacy concerns.

**Methods:**

This feasibility study was conducted in four urban, high abortion caseload facilities. Women enrolled in the study were randomized into an intervention (n=60) or control group (n=60) using block randomization. Women completed a baseline interview on the day of their abortion procedure and a follow-up interview 4 months later (retention rate: 89.1%, 107/120). Women in the intervention group received text message reminders to use their selected postabortion contraceptive methods and reminders to contact the facility if they had problems or concerns with their method. Women who did not select a method received weekly messages that they could visit the clinic if they would like to start a method. Women in the control group did not receive any messages.

**Results:**

Almost all women in the feasibility study reported using their mobile phones at least once per day (98.3%, 118/120) and 77.5% (93/120) used their phones for text messaging. In the intervention group, 87% (48/55) of women were using modern contraception at the 4-month follow-up, whereas 90% (47/52) were using contraception in the control group (*P*=.61). The intervention was not effective in increasing modern contraceptive use at follow-up, but 93% (51/55) of women reported at follow-up that the text reminders helped them use their method correctly and 76% (42/55) said they would sign up for this service again. Approximately half of the participants (53%, 29/55) said that someone they did not want to know about the text message reminders found out, mostly their husbands or children.

**Conclusions:**

In this small-scale feasibility study, text reminders did not increase postabortion contraceptive use. Despite the ineffectiveness of the text reminder intervention, implementation of a mHealth intervention among abortion clients in urban Bangladesh was feasible in that women were interested in receiving follow-up messages after their abortion and mobile phone use was common. Text messages may not be the best modality for a mHealth intervention due to relatively low baseline SMS text message use and privacy concerns.

## Introduction

Mobile technology has potential applications in many aspects of health, and studies have begun to explore how mobile health (mHealth) interventions can be used to support women to use their contraceptive method correctly and consistently to prevent unwanted pregnancy [1]. A recent study in the United States showed that providing complete and accurate information on contraceptives using a mHealth platform can be as effective as in-person counseling, allowing for patients to choose an effective method and helping maximize the use of in-person counseling [2]. A review of studies, mostly conducted in high-income countries, indicates that short message service (SMS) text messaging improves outcomes in antiretroviral therapy and smoking cessation interventions [3]. These studies suggest that SMS text messages may be beneficial as appointment reminders, but not for medication adherence [3]. Yet, studies in several African countries indicate acceptability of contraceptive and medication abortion information via SMS text message [4-6]. A study in rural Uganda testing an intervention with people living with HIV suggests promising results for using SMS text messages to reach rural and low-literacy populations and protecting patient privacy [7]. Nevertheless, a recent review of the utilization of mobile phone technology for improving contraceptive use concluded that there is insufficient evidence for promoting this technology and that the benefits of any intervention greatly depend on the components of the intervention [8]. Further documentation of efforts in a variety of settings, as well as acceptability among different populations, is needed.

The majority of wireless subscribers live in low- and middle-income countries [9]. Bangladesh, like many other low-income countries, has seen an increase in the population’s access to mobile technology, with 97% of the population having access to a mobile signal according to the Bangladesh Telecommunications Regulatory Commission [10]. Moreover, a study of household ownership of mobile phones indicates that, once the market was saturated, even households with low socioeconomic levels were able to become mobile owners [10]. As early as 2007, the Ministry of Health and Family Welfare recognized the potential of mHealth and has used mobile technology to broadcast health messages to all cell phone users [9]. Furthermore, a recent study on the impact of electronic media on contraceptive use in Bangladesh shows the promise of digital communication to improve use and continuation of contraceptives [11].

Bangladesh has a high pregnancy termination rate at 37 per 1000 women of reproductive age, compared to the average for South Asia at 26 per 1000 women [12,13]. Improving effective use of contraception after an abortion procedure is essential to reducing subsequent unwanted pregnancies. Abortion service statistics from Bangladesh demonstrate that the most prevalent form of contraception among postabortion clients (>80%) are short-acting methods. Oral contraceptive pills account for the majority of the methods (47%), followed by injectables at 20% and condoms at 15%. In the general population, there is a high rate of discontinuation among modern contraceptive users. Data from the 2011 Bangladesh Demographic and Health Survey indicate that 47% of condom acceptors, 39% of oral contraceptive pill acceptors, and 36% of injectable acceptors discontinue use within 12 months of initiation [14]. Local beliefs, such as the need to take periodic “breaks” from oral contraceptive pill use [15], may contribute to high discontinuation rates and the high rate of pregnancy termination in Bangladesh. Interventions to support contraceptive continuation are needed, perhaps especially among abortion clients who may have a history of inconsistent contraceptive use.

This study examines the feasibility of implementing a SMS text message-based mHealth intervention among abortion clients in urban Bangladesh to support contraceptive continuation among abortion clients who accept short-acting postabortion contraceptive methods and to promote contraceptive uptake among those who did not select a method. The project used method-specific text message reminders based on the contraceptive method the woman selected on the day of her abortion procedure. We report on mobile phone usage, satisfaction, and privacy concerns associated with the intervention.

## Methods

### Setting and Participants

This prospective study recruited 120 women from four urban sexual and reproductive health clinics run by the Reproductive Health Services Training and Education Program (RHSTEP) in the divisional capitals of Dhaka, Chittagong, Rajshahi, and Sylhet. Women were eligible for study participation if they received abortion services, selected a short-acting postabortion contraceptive method or no method on the day of their abortion procedure, did not intend to become pregnant in the next four months, did not intend to use their selected method as a temporary method (eg, using condoms temporarily while waiting for sterilization), and had a personal mobile telephone that used Global System for Mobiles (GSM) technology. Participants were randomized into the intervention or control group using computer-generated block randomization. The intervention group (n=60) received the full schedule of method-specific text reminders described subsequently and the control group (n=60) received no reminder messages.

### Intervention

Women in the intervention group received method-specific text message reminders to use their selected method (Table 1). The mobile phone number, woman’s preferred language, time of day to receive the reminders, and selected contraceptive method (pill, injectable, condom, or none) were entered into a Web-based platform. Data collectors were equipped with netbooks and entered women’s data into the system at the time of study enrollment. A test reminder was sent to the woman’s phone after completion of the baseline interview to ensure that the system was working properly and that women knew what to expect. Each message included a study number that participants were instructed to call if they wished to withdraw from the study. The intervention was provided at no cost to women because receiving text messages in Bangladesh is free. All women participating in the study received the standard abortion and postabortion contraceptive care available in the RHSTEP clinics.

**Table 1:** Schedule and content of text reminders by contraceptive method selected.

Method selected and frequency of messages		Content
**Pills**		
	Daily	Remember to take your medication.
	Weekly	Some women experience difficulties with their method. If you are having problems or have any questions about your method, please contact the clinic.
**Injectables**		
	One week before due date of next injection and on injection due date	You can return to the clinic to get your next injection.
	Weekly	Some women experience difficulties with their method. If you are having problems or have any questions about your method, please contact the clinic.
**Condoms**		
	Twice weekly	Remember to use your method.
	Weekly	Some women experience difficulties with their method. If you are having problems or have any questions about your method, please contact the clinic.
**None**		
	Weekly	You can visit the clinic if you would like to start a method.

### Data Collection

Baseline data collection took place from March to June 2013. After obtaining written informed consent, participants completed a 30-minute interviewer-administered survey conducted in Bangla on the day of their abortion procedure. The survey included women’s sociodemographic characteristics, fertility intentions, and their frequency of mobile phone usage. All respondents were asked to complete a follow-up survey 4 months after their procedure. Up to three attempts were made to contact each participant to schedule their follow-up interview, and the retention rate was 89.1% (107/120). In the follow-up interview, women in the intervention group were asked about their satisfaction with the intervention and any privacy concerns related to receiving SMS text message reminders. Follow-up data collection occurred from July to October 2013. All data were collected by trained female interviewers from a locally contracted nongovernmental organization, the Bangladesh Association for Prevention of Septic Abortion.

### Data Analysis

Completed questionnaires were checked for quality and consistency by research officers and entered into EpiData 3.1 software. The primary outcome of interest was modern contraceptive use at 4-month follow-up, which was assessed for the intervention and control group and compared using a chi-square test. Sociodemographic characteristics of women participating in the study, intervention preferences, and experiences with the SMS text message reminder intervention are presented. Percentages are computed among nonmissing responses. Data were analyzed using Stata/SE 14.0.

### Ethical Approval

Institutional Review Board approval was obtained from the Bangladesh Medical Research Council and Allendale Institutional Review Board in the United States.

### Intervention Development

A SMS text message system was designed and developed with technical assistance from a local information technology company, Iris Technology Bangladesh. The system used a Web-based platform with secured log-in and delivery to groups of clients depending on their selected postabortion contraceptive method and intervention preferences. Women were able to select their preferred time of day (morning, afternoon, or night) to receive the messages to improve privacy. For example, women could select to have the messages sent during the afternoon if they would be at home alone during that time. Women were also able to select their preferred language for the messages, including Bangla (Unicode), English, or phonetic Bangla in English fonts. The platform supported all GSM phones (used by more than 90% of Bangladesh Telecom subscribers) [16,17], but did not support Code Division Multiple Access phones. The system cost approximately US $2500, including design, customization, and registration. The cost of maintaining the system would be US $192 per year to maintain technical support from Iris Technology and US $0.04 per SMS text message sent.

## Results

### Characteristics of Study Participants

All 120 women in the sample were married and two-thirds (79/120) had one to two children (Table 2). In all, 85.8% (103/120) reported having secondary or higher education. Although 70.0% (84/120) of participants reported no financial difficulties, this differed by group assignment with 82% (49/60) of women in the intervention group and only 58% (35/60) of women in the control group reporting no financial problems (*P*=.005). Almost 78.3% (94/120) lived in an urban setting, as expected given the recruitment sites. A total of 44.1% (53/120) of participants selected pills as their postabortion contraceptive method, although 33.3% (40/120) selected injectables, 20.8% (25/120) selected condoms, and 1.7% (2/120) did not select a postabortion contraceptive method.

**Table 2:** Sociodemographic profile of study participants (N=120).

Sociodemographic characteristics		Total, n (%) (N=120)		Intervention, n (%) (n=60)		Control, n (%) (n=60)		*P*	
Woman’s age, mean (SD)		28.1 (5.9)		27.4 (5.8)		28.8 (6.0)		.19	
**Woman’s education, n (%)**								.13	
	None		4 (3.3)		0 (0)		4 (7)		
	Primary		13 (10.8)		7 (12)		6 (10)		
	Secondary or higher		103 (85.8)		53 (88)		50 (83)		
Husband’s age, mean (SD)		35.4 (7.6)		35.1 (7.9)		35.7 (7.4)		.68	
**Husband’s education, n (%)**								.44	
	None		6 (5.0)		2 (3)		4 (7)		
	Primary		11 (9.2)		4 (7)		7 (12)		
	Secondary or higher		102 (85.0)		53 (90)		49 (81)		
**Religion, n (%)**								.60	
	Islam		108 (90.0)		55 (92)		53 (88)		
	Hinduism		11 (9.2)		5 (8)		6 (10)		
	Buddhism		1 (0.8)		0 (0)		1 (2)		
**Marital status, n (%)**									
	Married		120 (100.0)		60 (100)		60 (100)		
**Number of children, n (%)**								.17	
	No children		14 (11.7)		10 (17)		4 (7)		
	1-2 children		79 (65.8)		39 (65)		40 (67)		
	≥3 children		27 (22.5)		11 (18)		16 (26)		
**Financial situation, n (%)**								.005	
	Difficult		36 (29.2)		11 (18)		25 (42)		
	Have no problems		84 (70.0)		49 (82)		35 (58)		
**Residence, n (%)**								.66	
	Urban		94 (78.3)		48 (80)		46 (77)		
	Rural		26 (21.7)		12 (20)		14 (23)		
**Postabortion contraceptive selected, n (%)**								.59	
	Pills		53 (44.2)		27 (45)		26 (43)		
	Injectables		40 (33.3)		17 (28)		23 (38)		
	Condoms		25 (20.8)		15 (25)		10 (17)		
	No method		2 (1.7)		1 (2)		1 (2)		

### Baseline Mobile Phone Use

Baseline mobile phone use was assessed among all 120 women enrolled in the study. Overall, 98.3% (118/120) of the women enrolled in the study reported using their mobile phone at least once per day. Although 77.5% (93/120) of women used their mobile phones for text messaging, the frequency of use was low. Approximately one-quarter (23.3%, 28/120) of women reported using text messaging every day, slightly more than one-third (34.2%, 41/120) used text messaging once a week and 20.0% (24/120 women) used text messaging less than once per week.

### Baseline Intervention Preferences and Readability of Messages

The majority of women in the intervention group (63%, 38/60) preferred phonetic Bangla in English fonts, primarily because their mobile phones did not support Bangla fonts. In all, 30% (18/60) selected Bangla (Unicode), and 7% (4/60) selected English as the language for their SMS text message reminders. Approximately three-quarters of participants (73%, 44/60) preferred to receive the messages in the evening, 23% (14/60) during the afternoon, and 3% (2/60) in the morning. At the time of study enrollment, a test message was sent to check readability and 93% (56/60) of women were able to read the full message in their selected language. All women were able to read at least part of the message.

### Intervention Effectiveness at Follow-Up

Intervention effectiveness was assessed at 4 months postabortion among the 89% (107/120) of women who completed the follow-up interview. Loss to follow-up was differential by education and financial status with poorer and less-educated women more likely to be lost to follow-up. A statistically significant difference in modern contraceptive use 4 months postabortion was not observed between intervention and control groups. In the intervention group, 87% (48/55) of women were using modern contraception at the time of the 4-month follow-up, whereas 90% (47/52) were using a method in the control group (*P*=.61).

### Satisfaction With the mHealth Intervention at Follow-Up

**Figure 1:**
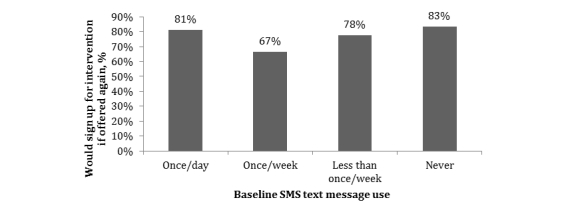
Interest in signing up for the intervention if offered again by baseline SMS text message use (n=55).

Women in the intervention group were asked about their satisfaction with the text reminders at follow-up. Overall, 93% (51/55) of women reported at follow-up that the text reminders helped them use their method correctly. Approximately 87% (48/55) of women said they were satisfied with the timing of the text message reminders and 84% (46/55) said they read the messages regularly. Approximately three-quarters (76%, 42/55) of women said they would sign up for the service again. Women who used their phones for SMS text messages regularly (81%, 13/16) and those who did not use their phones for SMS text messaging at baseline (83%, 10/12) were most likely to say that they would sign up again, whereas women who used their phones for SMS text messaging infrequently were less likely to be interested in signing up again (Figure 1). Among the 24% (13/55) who would not sign up for the service again, approximately half (n=5) said they did not need the reminder, two of the women said their husband did not like the SMS text message service, and one woman expressed privacy concerns.

### Privacy Concerns

**Figure 2:**
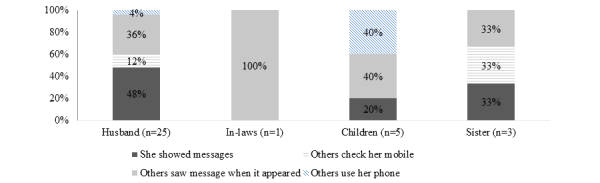
Ways in which family members found out about text messages (n=29).

Women were ineligible for study participation if they reported during the consent process that they shared their phone with someone else. Despite this precaution and asking women to select the time of day they wished to receive messages, some women reported challenges with privacy of text messages at follow-up. The majority of participants (93%, 51/55) reported that they were satisfied with the privacy of receiving text message reminders, but approximately half of participants (53%, 29/55) responded affirmatively to the statement “someone I did not want to know about the text reminders found out.” For most women (86%, 25/29), it was her husband who found out about the messages, for five women it was her children who found out, for three it was her sister, and for one woman, it was her in-laws who found out. When women were asked further questions about how another person found out, almost half of women who reported that their husbands found out (48%, 12/25) said they showed the message themselves, more than one-third (36%, 9/25) said their husband saw it when it appeared on their phone, and 12% (3/25) reported their husband found it when he checked her phone (Figure 2). For children, 40% (2/5) saw the message as it appeared and 40% (2/5) saw it when they were using her phone. Three women said that they were dissatisfied with the privacy of the text messages, and all three women reported that their children saw the messages and two also reported that their husband saw the messages. Although there were concerns about privacy of the SMS text message reminders, the majority of women reported communicating with their husband about participation in the study (91%, 50/55) and about the contraceptive method they chose after the abortion procedure (96%, 53/55) *.*

### Suggestions for Improving mHealth Intervention

In the follow-up interview, participants were asked to provide suggestions for improving the SMS text message service. Of 27 participants who offered their suggestions, approximately half (14/27) indicated that automated voice messages or phone call reminders would be better for privacy. Two respondents suggested the traditional mode of interpersonal communication through courtyard meetings. One respondent suggested having a number to call to get information. When asked specifically about interest in a hotline for information on abortion and contraception, 96% (53/55) of intervention participants responded that they would be interested in using a hotline. Being able to talk to a counselor was important to some women in the study. Participants were given a mobile phone number enabling them to call the researchers to withdraw from the study if they wanted, but according to call log records, 13% of participants (8/60) called the number with questions on contraceptive methods, although the number was not meant for this purpose. Women were also asked if they would answer questions using mobile text messaging if that incurred a cost. In response, 82% (45/55) of women reported that they would answer text messages if the cost was within the limit of 0.5 to 1.5 Taka (<US $0.01) per message.

## Discussion

The simple text reminder intervention did not increase postabortion contraceptive use 4 months postabortion. Despite the ineffectiveness of the text reminder intervention, this study has important findings for those seeking to implement mHealth interventions in the potentially vulnerable population of women receiving abortion services. This study finds that it is feasible to conduct a mHealth intervention with abortion clients in urban Bangladesh because mobile phone use was common and women were interested in receiving follow-up messages after their abortion. However, women also raised privacy concerns associated with text messages appearing on their phones unexpectedly. Reaching out to urban abortion clients through mobile phones could be a feasible strategy, but the best modality should be explored through additional formative work and privacy concerns addressed.

Approximately 93% (51/55) of women reported that the mobile text message helped them use their contraceptive method correctly, but the intervention had no effect on modern contraceptive use at 4 months postabortion. Previous studies have shown that mobile messaging can be a simple and effective means of supporting women’s access to sexual and reproductive health information and services [18,19]. However, the evidence for mobile health interventions’ effect on actual service utilization is variable, indicating the need for further rigorous analysis on mHealth’s contributions to long-term behavior change [8]. It is likely that more interactive and adaptive interventions have a greater impact on behavior change, compared to the simple reminder intervention tested in this study.

There was interest from participants in this study in having a hotline number that they could call with questions (96%, 53/55). Even though a majority of participants reported using text messaging, regular use was low with only 23% (28/120) using their phone for SMS text messaging at least once per day, and verbal communication may be preferred. Interest in signing up for the intervention again varied by SMS text message use at baseline, suggesting that women who already use SMS text messages regularly and those who have little experience with SMS text messages (and may find it novel), might be more interested in receiving SMS text messages than others. A hotline option might be acceptable to a broader group of women and would possibly increase privacy because women could call the hotline at their convenience. Some studies have suggested that interventions using verbal communication increase contraceptive uptake and use [8] and, in Cambodia, using voice messages that link abortion clients with a call center counselor demonstrated increased uptake of long-acting contraceptive methods in the months following abortion [20]. Although the more labor-intensive strategy of employing call center counselors to support women’s postabortion contraceptive use appears to be beneficial, the long-term cost-effectiveness is yet to be studied [8]. Additional work is needed to understand whether verbal communication, through automated voice messages or connecting women with a call center counselor, is more acceptable to women.

There were some concerns about privacy related to this intervention, and more work is needed to fully understand women’s concerns and preferences. Although 93% (51/55) of women said they were satisfied with the privacy of the SMS text message reminders, approximately half (29/55) reported that someone they did not want to find out about the SMS text message reminders found out. These findings point to the importance of asking multiple questions related to privacy and not relying only on reports of satisfaction with the privacy that a mHealth intervention affords. When women were further asked how the person found out, most women reported showing the message themselves. The nuanced findings related to self-initiated sharing of text messages may suggest that mHealth interventions could contribute to increased communication about contraception between women and their husbands or partners. Studies have shown that mHealth interventions can lead to improved communication about sexual and reproductive health among partners and to positively influence gender dynamics through increased male cooperation and involvement in health areas that are normally the domain of the woman [21]. However, it is also possible that the women felt obligated to show the message so that no one would be suspicious regarding their communications or were pressured to show the message. The unified theory of acceptance and use of technology provides a useful framework for assessing how characteristics such as gender, age, and social influence impact use of technology. Our findings suggest that in this population, husbands and other family members impact women’s use of technology, especially when receiving information on potentially sensitive subjects such as contraception. Women’s privacy may be better protected through use of voice calls compared to SMS text messages [18], and future research on similar mHealth interventions should carefully assess how intervention modality can address women’s privacy concerns.

### Limitations

The feasibility of using the mHealth intervention with abortion clients should be judged within the context of this specific study sample. The study was conducted only in urban areas and findings may not generalize to rural areas of Bangladesh. Further investigation of mobile phone use in rural areas can provide insight into the feasibility of this type of intervention throughout the country. In addition, the small sample size limits power to detect changes in postabortion contraceptive use between baseline and follow-up. Women in the intervention group were wealthier than those in the control group and this baseline imbalance between the two groups could contribute to differences in contraceptive outcomes at follow-up. In addition, poorer and less-educated women were more likely to be lost to follow-up, which could result in an overestimate of postabortion contraceptive use at follow-up. Finally, we lack data on technologic aspects of the intervention, such as the message success rate, and on provider opinions about the intervention, which would have provided more information on the feasibility of conducting a mHealth intervention in this population.

### Conclusions

Women’s interest and satisfaction with the intervention suggests that a mHealth intervention to support postabortion contraceptive use is feasible in this context, but a more interactive intervention may be required to influence women’s postabortion contraceptive outcomes. In addition, SMS may not be the best intervention modality. Abortion clients in urban Bangladesh regularly use their mobile phones, but mobile use for SMS text messages is less common. In addition, lack of privacy of text messages raises concerns, and it may be more acceptable and effective to share information through an intervention that uses a voice messaging system or connects women with a call center counselor to allow for two-way conversation. Additional formative research is needed to customize the modality and content of a mHealth intervention that will be effective in supporting women’s postabortion contraceptive use.

## Abbreviations

GSM: Global System for Mobiles
RHSTEP: Reproductive Health Services Training and Education Program
SMS: short message service
